# Outcome of lung transplantation in patients with pulmonary alveolar microlithiasis in the era of COVID-19 infection

**DOI:** 10.1093/jscr/rjae211

**Published:** 2024-04-10

**Authors:** Lekhya Raavi, Pankaj Garg, Mohammad Alomari, Nafiye B Celik, Ian A Makey, Mathew Thomas, Aziza Nassar, Basar Sareyyupoglu, Samuel Jacob, Si M Pham, Magdy M El-Sayed Ahmed

**Affiliations:** Department of Cardiothoracic Surgery, Mayo Clinic, 4500 San Pablo Road, Jacksonville 32224, FL, United States; Department of Cardiothoracic Surgery, Mayo Clinic, 4500 San Pablo Road, Jacksonville 32224, FL, United States; Department of Cardiothoracic Surgery, Mayo Clinic, 4500 San Pablo Road, Jacksonville 32224, FL, United States; Department of Cardiothoracic Surgery, Mayo Clinic, 4500 San Pablo Road, Jacksonville 32224, FL, United States; Department of Cardiothoracic Surgery, Mayo Clinic, 4500 San Pablo Road, Jacksonville 32224, FL, United States; Department of Cardiothoracic Surgery, Mayo Clinic, 4500 San Pablo Road, Jacksonville 32224, FL, United States; Department of Pathology, Mayo Clinic, 12220 Kinneil Court, Jacksonville, FL 32224, United States; Department of Cardiothoracic Surgery, Mayo Clinic, 4500 San Pablo Road, Jacksonville 32224, FL, United States; Department of Cardiothoracic Surgery, Mayo Clinic, 4500 San Pablo Road, Jacksonville 32224, FL, United States; Department of Cardiothoracic Surgery, Mayo Clinic, 4500 San Pablo Road, Jacksonville 32224, FL, United States; Department of Cardiothoracic Surgery, Mayo Clinic, 4500 San Pablo Road, Jacksonville 32224, FL, United States; Department of Surgery, Zagazig University Faculty of Medicine, Koliat Altob st., Zagazig 44519, Egypt

**Keywords:** lung transplantation, pulmonary alveolar microlithiasis, COVID-19

## Abstract

Lung transplant recipients are at higher risk of developing COVID-19 infection compared to other solid organ transplants. The risk further increases in the unvaccinated patients. We present a case of a 43-year-old male who underwent bilateral sequential lung transplantation for pulmonary alveolar microlithiasis (PAM) and had an uneventful recovery. However, two years post-transplantation, the patient developed chronic lung allograft dysfunction (CLAD) with bronchiolitis obliterans syndrome and two episodes of COVID-19 infection. During the second episode of COVID-19 infection, the patient developed sepsis and multi-organ dysfunction ultimately resulting in death. Our case report highlights the increased susceptibility of PAM patients’ post-lung transplant to COVID-19 infection. Continuous follow-up of PAM patients’ post-lung transplantation is necessary to prevent unfavorable outcomes.

## Introduction

**Figure 1:**
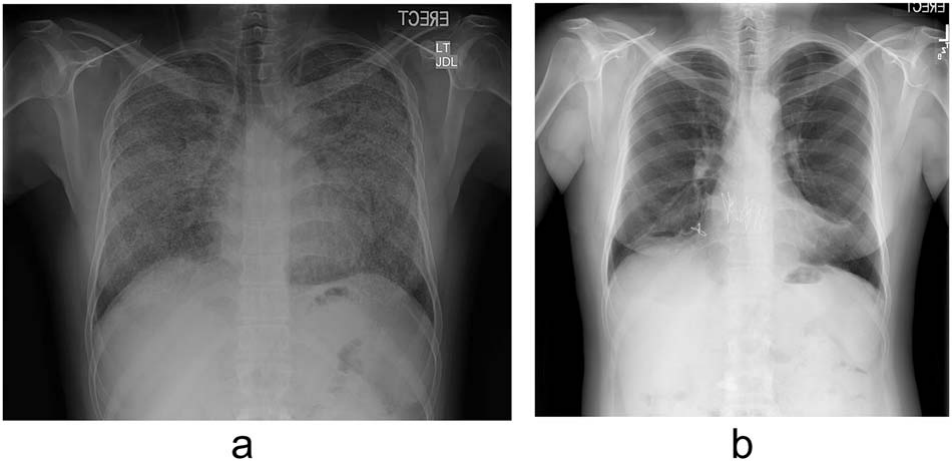
(a) Chest radiograph shows extensive calcification all over both lungs’ fields. (b) Chest radiograph after the transplant.

Pulmonary alveolar microlithiasis (PAM) is a rare autosomal recessive disease that develops due to the mutations of the ‘solute carrier family 34 member 2’ (SLC34A2) gene”. This results in the deposition of calcium phosphate crystals [1]. The only proven definitive treatment for PAM is lung transplantation (LTx) [2]. Herein, we report a patient of PAM who subsequently developed secondary severe pulmonary hypertension (PH) and polycythemia and received a bilateral sequential LTx (Fig. 1). His course during follow-up was complicated by chronic lung allograft dysfunction (CLAD) and two episodes of COVID-19 infection resulting in death of the patient 3 years after the transplant. We will also review the outcome of patients with PAM after LTx and the effect of COVID-19 infection on recipients of LTx.

## Case report

A 43-year-old Middle Eastern male, former smoker referred to our institute with chronic respiratory failure due to PAM for 5–6 years and oxygen dependence for 2 years and was listed for LTx. On investigations, patient had severe PH and moderate to severe right ventricle (RV) dilation and moderate RV dysfunction. His family history was negative for PAM. Pulmonary function test demonstrated forced expiratory volume in the first second (FEV1) 53%, forced vital capacity (FVC) 47%, total lung capacity (TLC) 47%, and diffusing capacity of the lungs for carbon monoxide (DLCO) 22% of predicted. His chest X-ray (Fig. 1a) was consistent with PAM. A donor became available, and patient underwent bilateral sequential LTx through clam-shell incision on veno-arterial extracorporeal membrane oxygenation (VA-ECMO) in September 2018 (Fig. 1b). Explanted lungs demonstrated anthracotic pleural surface with diffuse fibrous adhesions. Upon sectioning, lung parenchyma showed diffuse fibrosis and bilateral diffuse calcifications. Histopathology was also consistent with PAM. His postoperative course was complicated by primary graft dysfunction requiring VA-ECMO support for 2 days, prolonged mechanical ventilation with tracheostomy, right hemidiaphragm paralysis managed conservatively, and right femoral vein thrombosis requiring inferior vena cava filter insertion. Patient was discharged on post-operative Day 30 after decannulating the tracheostomy. Post-discharge, patient did well without any features of rejection on biopsy. Patient could not follow-up from January 2020 till June 2021 due to travel restrictions and had relatively poor compliance with immunosuppression due to unavailability. Patient had one episode of COVID-19 infection in April 2021 requiring hospitalization and had uneventful recovery. In June 2021, patient presented to our institute with worsening dyspnea and was diagnosed with CLAD with bronchiolitis obliterans syndrome and restrictive allograft syndrome without acute lung rejection (grade 0) and C4d immunostaining. However, donor specific antibodies were positive for HLADQ7 with mean fluorescence intensity (MFI) >5000. Pulmonary function test demonstrated FEV1 42%, FVC 53%, TLC 62%, and DLCO 40% of predicted. The patient was treated with intravenous immunoglobulin and underwent five cycles of plasmapheresis. Repeat donor specific antibody MFI reduced to below 5000 and lung biopsies remained negative for acute or chronic rejection. There was no change in FEV1 and FVC. There was no recurrence of calcification in the transplanted lungs. Patient was discharged with home oxygen therapy and maintenance intravenous immunoglobulin monthly for 6 months. In December 2021, patent again developed COVID-19 pneumonia that worsened, and patient was managed with mechanical ventilation and anti-viral medications. However, patient succumbed to death in January 2022 due to sepsis and multi-organ failure 28 days after hospitalization.

## Discussion

PAM is slowly progressive debilitating disease without any proven medical therapy and patients with end-stage lung disease are best managed by orthotopic lung transplant [3]. Due to rarity of disease, there are no well-defined criteria for LTx in these patients [4]. Similar to other lung pathologies, LTx should be considered in patients who become oxygen dependent, develop recurrent pneumothorax, or have recurrent episodes of acute exacerbations. [5] Further, to improve the outcome, LTx should be considered before the development of significant RV dysfunction. [6] Furthermore, the PAM results in the development of significant intrapulmonary shunting. Therefore, double LTx whenever possible is better than single lung transplant although there have been reports of successful outcome after single LTx. As the PAM only affects the lungs, LTx is usually curative [2].

### Outcome of lung transplant in patients with PAM in the era of COVID

Supplementary Table 1 summarizes the survivors after double or single LTx for end-stage PAM while Supplementary Table 2 summarizes the patients who died after LTx including our patient who died 39 months after double LTx due to COVID-19 pneumonia. Total 23 patients have undergone LTx for PAM with 82.6% (19 patients) survival. All the four patients who died underwent bilateral LTx. One patient died on POD 5 due to persistent bleeding while, the other patient died on POD11 due to sepsis. Third patient died at 3 months after LTx due to sepsis. Our patient died 39 months after the transplant due to recurrent COVID-19 infection. Ours is the first patients who died due to COVID-19 infection after LTx for PAM. Our patient developed CLAD during follow-up due to irregular immunosuppressive medication intake. Further patient developed two episodes of COVID infection. Patient made an uneventful recovery from the first episode however, during the second episode of COVID pneumonia, patient died due to cardiogenic shock, sepsis, and multiorgan failure.

In LTx recipients, mortality due to COVID infection remains significantly high compared to general population and other solid organ transplant recipients. Mortality rate in these patients is 34% and higher if mechanical ventilation is required [6–8].

Although less common, LTx recipients may also acquire second episode COVID infection as in our patient. Permpalung *et al.* [9] reported an increased risk of subsequent COVID infections and re-hospitalization within 90 days. The risk of morbidity in terms of decreased exercise capacity and DLCO, and mortality is further increased by presence of CLAD in transplanted lung as was seen in our patient [10].

## Conclusion

Lung transplant patients are at the increased risk of COVID-19 infection due to immunosuppression. These patients should be closely monitored to prevent unfavorable outcomes. Due to the limited PAM cases and literature on the lung transplantation post COVID-19, further extensive studies are required to observe the effect of COVID-19 on PAM lung transplanted patients.

## Supplementary Material

Supplementary_tables_rjae211
