# Supplementary material for: Outcome of lung transplantation in patients with pulmonary alveolar microlithiasis in the era of COVID-19 infection
Source: J Surg Case Rep. 2024 Apr 10;2024(4):rjae211. doi: 10.1093/jscr/rjae211 (PMC11008913; doi:10.1093/jscr/rjae211)
Supplement: Supplementary_tables_rjae211 [file supplementary_tables_rjae211.pdf]

Supplementary table 1: Survivors after double or single lung transplantation for end-stage PAM

References/Year	Age	Sex	Family history	Double/Single	Side	Preoperative condition	Complications	Follow-up(months)
Bonnette et al [1] 1992	46	NR	NR	Double	-	NR	Hemodynamic instability due to hilar dissection	NR
Stamatis et .al [2] 1993	32	M	Yes	Double	-	Severe pulmonary hypertension, right ventricular hypertrophy	Major bleeding	18
Raffa et al [3] 1996	47	F	NR	Single	Left	Severe pulmonary hypertension	Acute rejection, Bronchial stricture, and stenosis	12
Edelman et al [4] 1997	35	M	NR	Double	-	Spontaneous pneumothorax, pleurectomy	Uneventful	32
Jackson et .al [5] 2001	53	F	NR	Single	Right	NR	Uneventful	90
Shadmehr et al [6] 2009	32	M	NR	Single	NR	NR	Hemodynamic instability, reperfusion pulmonary edema	60
Shigemura et .al [7] 2010	63	F	NR	Double	-	Spontaneous pneumothorax	Uneventful	16
Samano et al [8] 2010	47	M	No	Double	-	H/o TB, received treatment Pulmonary hypertension	Hemodynamic instability, Pulmonary reperfusion syndrome, Distributive shock, Acute renal failure, on dialysis for 26 days	12
Borrelli et al [9] 2013	64	F	NR	Single	Right	NR	Uneventful	60
Güçyetmez et .al[10] 2014	52	F	NR	Double	-	NR	NR	12

Klikovits et .al [11] 2016	52	F	NR	Double	-	NR	Reperfusion -edema, atrial fibrillation, Intraoperative ECMO support	74
	34	M			-		Intraoperative ECMO, surgery uneventful	
	52	F			-		Intraoperative ECMO, surgery uneventful	
	62	F			-		Intraoperative ECMO, Atrial fibrillation	
Delic et al [12] 2016	73	F	No	Double	-	NR	NR	NR
Ren et al [13] 2019	52	M	NR	Single	Left	No	Bronchial anastomotic stenosis Chronic bacterial colonization	12
Jindal et al [14] 2019	54	F	NR	Double	-	No	No	12
Alrossais et al [15]2019	49	M	No	Double	-	Pulmonary hypertension Polycythemia	No	0.25
Helmink et al [16] 2021	48	M	Yes	Double	-	No	Multiple readmissions Acute rejection Sternal dehiscence	NR

M- Male, F- Female, NR- Not recorded, TB – Tuberculosis, ECMO - Extracorporeal membrane oxygenation

Supplementary table 2: Patients who died after lung transplantation

References /Year	Age	Sex	Family history	Double/Single lung transplantation	Preoperative condition	Cause of death	Outcome
Edelman et al [4] 1997	56	M	No	Double	Several spontaneous pneumothoraxes subsequently resulting in progressive cor pulmonale, Ascites, Hepatomegaly, Anasarca	Significant blood loss, hemodynamic instability and progressive hypoxemia	5d
Coulibaly et .al [17] 2009	49	F	NR	Double	NR	Infection	3 months
Klikovits et .al [11] 2016	32	F	NR	Double	NR	Primary graft dysfunction, Sepsis, intra and post operative ECMO	11d
Index Case 2021	46	M	No	Double	No	Acute graft dysfunction, inotropic support, VA- ECMO	39 months

M- Male, F- Female, NR – Not recorded

References:

1. Bonnette P, Bisson A, el Kadi NB, Colchen A, Leroy M, Fischler M, Loirat P, Caubarere I. Bilateral single lung transplantation. Complications and results in 14 patients. Eur J Cardiothorac Surg. 1992;6(10):550-4. doi: 10.1016/1010-7940(92)90007-k. PMID: 1389238.
2. Stamatis G, Zerkowski HR, Doetsch N, Greschuchna D, Konietzko N, Reidemeister JC. Sequential bilateral lung transplantation for pulmonary alveolar microlithiasis. Ann Thorac Surg. 1993 Oct;56(4):972-5. doi: 10.1016/0003-4975(93)90370-w. PMID: 8215680.
3. Raffa H, El-Dakhakhny M, Al-Ibrahim K, Mansour MS. Single lung transplantation for alveolar micro-lithiasis: the first clinical report. Saudi J Kidney Dis Transpl. 1996 Apr-Jun;7(2):189-93. PMID: 18417939.
4. Edelman JD, Bavaria J, Kaiser LR, Litzky LA, Palevsky HI, Kotloff RM. Bilateral sequential lung transplantation for pulmonary alveolar microlithiasis. Chest. 1997 Oct;112(4):1140-4. doi: 10.1378/chest.112.4.1140. PMID: 9377936.
5. Jackson KB, Modry DL, Halenar J, L'abbe J, Winton TL, Lien DC. Single lung transplantation for pulmonary alveolar microlithiasis. J Heart Lung Transplant. 2001 Feb;20(2):226. doi: 10.1016/s1053-2498(00)00500-3. PMID: 11250426.
6. Shadmehr MB, Arab M, Pejhan S, Daneshvar A, Javaherzadeh N, Abbasi A, Ahmadi ZH, Radpay B, Dabir S, Parsa T, Mohammadi F, Mansoori SD, Tabarsi P, Amiri MV, Marjani M, Kashani BS, Najafizadeh K, Shafaghi S, Ghorbani F, Masjedi MR, Velayati AA. Eight years of lung transplantation: experience of the National Research Institute of Tuberculosis and Lung Diseases. Transplant Proc. 2009 Sep;41(7):2887-9. doi: 10.1016/j.transproceed.2009.07.016. PMID: 19765464.

7. Shigemura N, Bermudez C, Hattler BG, Johnson B, Crespo M, Pilewski J, Toyoda Y. Lung transplantation for pulmonary alveolar microlithiasis. J Thorac Cardiovasc Surg. 2010 Mar;139(3):e50-2. doi: 10.1016/j.jtcvs.2008.07.066. Epub 2009 Feb 23. PMID: 19660326.
8. Samano MN, Waisberg DR, Canzian M, Campos SV, Pêgo-Fernandes PM, Jatene FB. Lung transplantation for pulmonary alveolar microlithiasis: a case report. Clinics (Sao Paulo). 2010 Feb;65(2):233-6. doi: 10.1590/S1807-59322010000200016. PMID: 20186308; PMCID: PMC2827711.
9. Borrelli R, Fossi A, Volterrani L, Voltolini L. Right single-lung transplantation for pulmonary alveolar microlithiasis. Eur J Cardiothorac Surg. 2014 Feb;45(2):e40. doi: 10.1093/ejcts/ezt531. Epub 2013 Nov 19. PMID: 24258203.
10. Güçyetmez B, Ogan A, Cimet Ayyıldız A, Yalçın Güder B, Klepetko W. Lung transplantation in an intensive care patient with pulmonary alveolar microlithiasis - a case report. F1000Res. 2014 May 28;3:118. doi: 10.12688/f1000research.4035.1. PMID: 25165536; PMCID: PMC4133765.
11. Klikovits T, Slama A, Hoetzenecker K, Waseda R, Lambers C, Murakoezy G, Jaksch P, Aigner C, Taghavi S, Klepetko W, Lang G, Hoda MA. A rare indication for lung transplantation - pulmonary alveolar microlithiasis: institutional experience of five consecutive cases. Clin Transplant. 2016 Apr;30(4):429-34. doi: 10.1111/ctr.12705. Epub 2016 Feb 24. PMID: 26841075.
12. Delic JA, Fuhrman CR, Trejo Bittar HE. Pulmonary Alveolar Microlithiasis: AIRP Best Cases in Radiologic-Pathologic Correlation. Radiographics. 2016 Sep-Oct;36(5):1334-8. doi: 10.1148/rg.2016150259. PMID: 27618319.

13. Ren XY, Fang XM, Chen JY, Ding H, Wang Y, Lu Q, Ming JL, Zhou LJ, Chen HW. Single-lung transplantation for pulmonary alveolar microlithiasis: A case report. World J Clin Cases. 2019 Nov 26;7(22):3851-3858. doi: 10.12998/wjcc.v7.i22.3851. PMID: 31799314; PMCID: PMC6887609.
14. Jindal A, Rahulan V, Balasubramani G, Dutta P, Attawar S. Pulmonary alveolar microlithiasis: A rare disease treated with lung transplantation, first case from India. Lung India. 2019 Nov-Dec;36(6):546-549. doi: 10.4103/lungindia.lungindia_50_19. PMID: 31670305; PMCID: PMC6852219.
15. Alrossais NM, Alshammari AM, Alrayes AM, Mohammad N, Al-Amoodi MJH, Almutairi AM, Alsuhaymi AO, Alhadid DA, Alhammad FA, Ouf NH, Ahmed MH, Saleh W, AlAmodi AA. Pulmonary Hypertension and Polycythemia Secondary to Pulmonary Alveolar Microlithiasis Treated with Sequential Bilateral Lung Transplant: A Case Study and Literature Review. Am J Case Rep. 2019 Jul 28;20:1114-1119. doi: 10.12659/AJCR.911045. PMID: 31352464; PMCID: PMC6683309.
16. Helmink A, Atiya S, Martinez Duarte E. Pulmonary Alveolar Microlithiasis: A Unique Case of Familial PAM Complicated by Transplant Rejection. Case Rep Pathol. 2021 Apr 5;2021:6674173. doi: 10.1155/2021/6674173. PMID: 33884208; PMCID: PMC8041554.
17. Coulibaly B, Fernandez C, Reynaud-Gaubert M, D'Journo X, Doddoli C, Taséi AM. Microlithiase alvéolaire avec fibrose interstitielle sévère conduisant à la greffe [Alveolar microlithiasis with severe interstitial fibrosis leading to lung transplantation]. Ann Pathol. 2009 Jun;29(3):241-4. French. doi: 10.1016/j.annpat.2009.02.015. Epub 2009 Jun 12. PMID: 19619834.
